# Synthesis and Biological Activity of Some Bile Acid-Based Camptothecin Analogues

**DOI:** 10.3390/molecules19033761

**Published:** 2014-03-24

**Authors:** Xingnuo Li, Tengfei Zhao, Dongping Cheng, Chu Chu, Shengqiang Tong, Jizong Yan, Qing-Yong Li

**Affiliations:** 1 College of Pharmaceutical Science, Zhejiang University of Technology, Hangzhou 310014, China; E-Mails: lixingnuo@zjut.edu.cn (X.L.); Chengdp@zjut.edu.cn (D.C.); chuchu@zjut.edu.cn (C.C.); sqtong@zjut.edu.cn (S.T.); yjz@zjut.edu.cn (J.Y.); 2 Key Laboratory of Forest Plant Ecology (Northeast Forestry University), Ministry of Education, 332# No. 26 Hexing Road, Harbin 150040, China; E-Mail: li_qingyong@163.com

**Keywords:** camptothecin, bile acids, anti-tumour activity, hepatoma cells

## Abstract

In an effort to decrease the toxicity of camptothecin (CPT) and improve selectivity for hepatoma and colon cancer cells, bile acid groups were introduced into the CPT 20 or 10 positions, resulting in the preparation of sixteen novel CPT-bile acid analogues. The compounds in which a bile acid group was introduced at the 20-hydroxyl group of CPT showed better cytotoxic selectivity for human hepatoma and colon cancer cells than for human breast cancer cells. Fluorescence microscopy analysis demonstrated that one compound (**E2**) entered human hepatoma cells more effectively than it did human breast cancer cells. Compound **G4** exhibited the best anti-tumour activity *in vivo*. These results suggested that introduction of a bile acid group at the 20-position of CPT could decrease toxicity *in vivo* and improve selectivity for hepatoma cells.

## 1. Introduction

The search for effective anti-cancer drugs has led to the identification of many compounds that effectively inhibit cancer cells. However, these cytotoxic agents usually have very poor specificity, and therefore cause systemic toxicity. Combining drugs with more specific carriers could improve targeting, increase the local drug concentration, improve cell penetration, and reduce drug toxicity. For these reasons, recent research has been focused on drug carriers.

Camptothecin (CPT) is a highly effective anti-tumour and antibiotic alkaloid that was first isolated by Wall and Wani in 1958 from the extracts of *Camptotheca acuminate*, a tree native to China [1,2]. CPT is a neutral alkaloid that easily reacts with acids to form salts and does not readily dissolve in acid solutions, water, or common organic solvents. Moreover, the CPT α-hydroxyl lactone E-ring is unstable, and undergoes rapid hydrolysis to an inactive carboxylate form at physiological pH [3]. The open loop of CPT leads to serious side effects, which led to the termination of clinical trials on CPT. By binding to DNA topoisomerase I (Topo I), CPT increases DNA damage and triggers apoptosis in rapidly multiplying cancer cells and also in normal cells containing high levels of Topo I. Analysis of CPT structure-activity relationships has indicated that modification of the 20-hydroxyl position of CPT could eliminate intramolecular hydrogen bonding and reduce carbonyl activity. Because this modification increases carbonyl resistance to a nucleophile attack, lactone E-ring analogues of CPT show increased stability and improved anti-tumour activity [1]. Introducing changes at the CPT 10-hydroxyl position also enhances lactone stability. Moreover, this method could enhance the drug’s anti-cancer activity by preventing CPT from binding with human serum albumin.

Some bile acids and their derivatives can be used as drug carriers. In the enterohepatic circulation, it enters the liver and colon cells by active transport. A large number of studies have shown that conjugating drugs with appropriate bile acid could increase their enterohepatic absorption, improve metabolic stability, enhance oral bioavailability, and reduce toxicity [4,5,6,7,8,9,10]. The latest research focuses on structure-activity relationships and the application of bile acid as a drug carrier. These studies have indicated that drugs coupled to the bile acid 3 and 24 sites did not affect the acid part of the activity [6,7], and the activities of most drugs were not affected by coupling to a bile acid [8]. Therefore, bile acids have the potential to be useful drug carriers [9].

The aim of the present study was to design a new tumour-targeting drug consisting of CPT as the cytotoxic warhead, and a bile acid as the tumour recognition moiety. We hypothesised that this could produce a new anti-tumour drug with liver and colon selectivity and reduced toxicity. We have previously synthesised CPT-bile acid analogues and evaluated their anti-tumour activities, and the research showed that bile acid linked to the 20-hydroxy position of CPT did not affect the anti-tumour activity of CPT [10]. The present study extended the above work by synthesising a series of CPT-bile acid analogues and investigating their anti-tumour activities *in vitro* and *in vivo*.

## 2. Results and Discussion

### 2.1. Chemistry

**Scheme 1:**
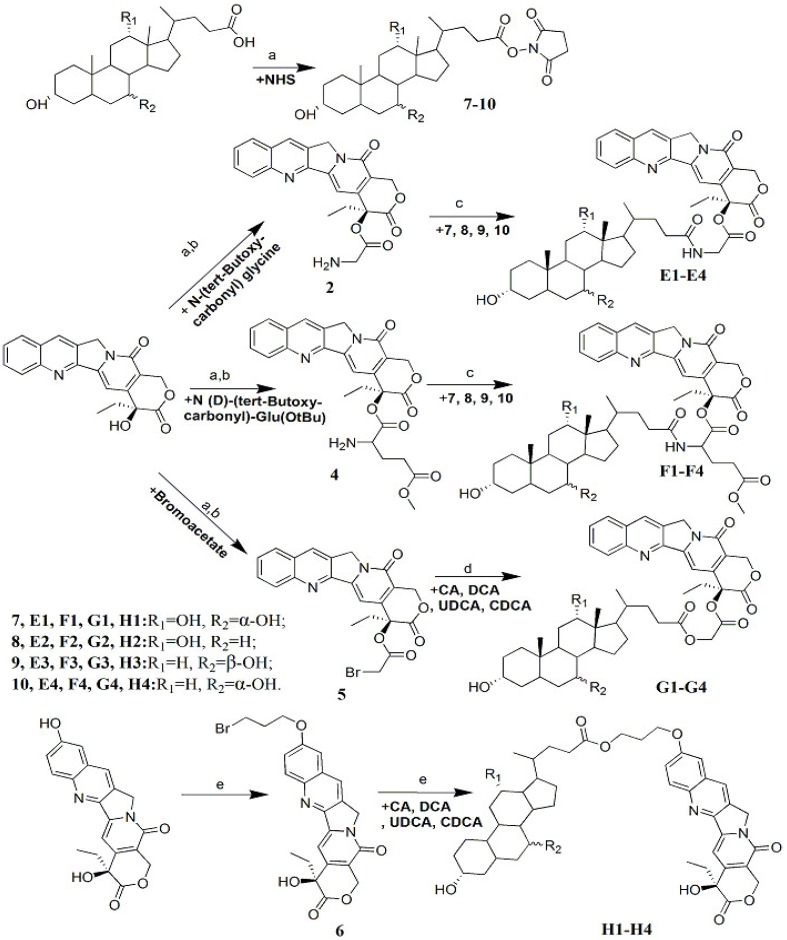
Synthesis of CPT-bile acid analogues.

In our study, a range of CPT-bile acid analogues were prepared using different bile acids (**E1**, **F1**, **G1**, **H1**: cholic acid, CA; **E2**, **F2**, **G2**, **H2**: deoxycholic acid, DCA; **E3**-, **F3**, **G3**, **H3**,: ursodeoxycholic acid, UDCA; **E4**-, **F4**, **G4**, **H4**, chenodeoxycholic acid, CDCA) and linkages, as shown in Scheme 1.

*Reagents*: (a) EDCI, DMAP, and CH_2_Cl_2_; (b) HCl/MeOH; (c) TEA and DMSO; (d) TEA and DMF; (e) K_2_CO_3_ and DMF. (CA = cholic acid; DCA = deoxycholic acid; UDCA = ursodeoxycholic acid; CDCA = chenodeoxycholic acid).

The target compounds **E1**–**E4** were synthesised by forming an amide connecting the bile acid with CPT. The CPT was mixed with *N*-(*tert*-butoxycarbonyl) glycine in chloroform, 1-[3-(dimethylamino) propyl]-3-ethylcarbodiimide hydrochloride (EDCI), and a catalyst, 4-dimethylaminopyridine (DMAP), at room temperature to yield compound camptothecin-20(*S*)-*O*-(*N*-(*tert*-butoxycarbonyl) glycine) ester (**1**) [11]; The amino group of compound **1** was deprotected in HCl/CH_3_OH to produce compound **2**, which was then attached to activated bile acids **7**–**10** in the presence of triethylamine to produce compounds **E1**–**E4** in yields of 75%–76%. The target compounds **F1**–**F4** were synthesised using the method described above for **E1**–**E4**, but the linker chain used was d-Glu(OMe) and the yields were 64%–66%. The target compounds **G1**–**G4** were synthesised by forming an ester bond connecting CPT and a bile acid. CPT was first converted into compound **5** at high yield (97.5%) in the presence of EDCI and DMAP at room temperature, and then the alkyl halide part of compound **5** underwent a nucleophilic substitution reaction with the bile acid carboxyl group in an alkaline environment, producing compounds **G1**–**G4** in 62%–63% yield. This reaction occurred faster in the presence of K_2_CO_3_, but more byproduct that could not be separated easily was produced. Use of triethylamine as the catalyst resulted in a slower reaction but fewer impurities. The target compounds **H1**–**H4** were synthesised via formation of an ester bond connecting the bile acid with 10-hydroxycamptothecine (HCPT). The HCPT hydroxyl group engaged in a nucleophile substitution reaction with 1,3-dibromopropane in dimethylsulphoxide (DMSO) at 60 °C in the presence of K_2_CO_3_, to produce compound **6** [12] Compound **6** underwent nucleophilic substitution reactions with bile acids in a similar manner to produce **H1**–**H4** in yields of 71%–73%. The proton nuclear magnetic resonance (^1^H-NMR), electrospray ionization-mass spectrometry (ESI-MS), and infrared (IR) spectra of these novel CPT derivatives helped confirm that all the products were consistent with their predicted structures.

### 2.2. Pharmacology

We tested the biological activities of these novel CPT-bile acid analogues *in vitro* and *in vivo*. We also studied their ability to enter cells and their stability in plasma. The *in vitro* anti-tumour activities of the CPT-bile acid analogues were evaluated against a human colon cancer cell line (HCT-116), a human hepatoma cell line (SMMC-7721), and a human breast cancer cell line (MCF-7). These studies resulted in the selection of compound **E2** for subsequent examination of its entry into SMMC-7721 and MCF-7 cells. In addition, the *in vivo* anti-tumour activities of compounds **E2**, **G4**, and **H2** against a mouse liver adenocarcinoma model (H_22_) were investigated. The stability of these novel CPT-bile acid derivatives in serum was measured using high-performance liquid chromatography (HPLC).

#### 2.2.1. MTT Test

The results of the *in vitro* anti-tumour studies are summarized in Table 1. These results showed that although CPT showed similar toxicity towards the three cell lines, and the cells showed different sensitivities to the novel analogues. The majority of the compounds were more toxic to the human colon cancer and hepatoma cell lines (HCT-116 and SMMC-7721) than to the human breast cancer cell line (MCF-7). This indicated selectivity of the prepared analogues with bile acids at the CPT 20-hydroxyl position for colon cancer and hepatoma cells in Table 1. With regard to the SMMC-7721 cell line, compound **G4** showed the highest cytotoxic activity (IC_50_ = 0.14 µM), four times more effective than CPT. For the HCT-116 cell line, compounds **H2** and **H3** showed the highest cytotoxicity (IC_50_ = 0.8 µM), similar to that of CPT. For the MCF-7 cell line, compound **H2** showed the highest cytotoxicity (IC_50_ = 2.5 µM), which was still much less effective than CPT (IC_50_ = 0.57). E series were generally more cytotoxic to the HCT-116 and SMMC-7721 cell lines according to Table 1. The activities of **G1**–**G4** and **E1**–**E4** showed that the use of amide or ester bonds did not affect the cytotoxic activity of the analogues. It is entirely possible that binding of bile acids at CPT’s 10-hydroxyl group contributed to decreased selectivity for colon cancer and hepatoma cells.

**Table 1:** Cytotoxicity of CPT-bile acid analogues.

Compounds	*In Vitro* Cytotoxicity (IC_50_, µM)			Half-Life (h)
	MCF-7 HCT-116 SMMC7721			
CPT	0.57	0.52	0.56	2.3
E1	18.3	2.9	2.23	NT
E2	150	15.8	0.36	>24
E3	61	2.4	2.18	NT
E4	20	2.88	2.25	NT
F1	40	70	55.7	NT
F2	11.8	36	2.25	>24
F3	46.3	13	12.2	NT
F4	120	20	2.23	NT
G1	10.9	4	0.71	NT
G2	4	2.05	1.24	>24
G3	62	12.1	6.84	NT
G4	70	36	0.14	NT
H1	3	7.4	3.2	NT
H2	2.5	0.8	2.74	>24
H3	2.8	0.8	3.34	NT
H4	30	1.4	2.18	NT

NT, not tested; MCF-7, human breast cancer cell line; HCT-116, human colon cancer cell line; SMMC-7721, human liver cancer cell line.

#### 2.2.2. Fluorescence and Phase Contrast Microscopy

**Figure 1:**
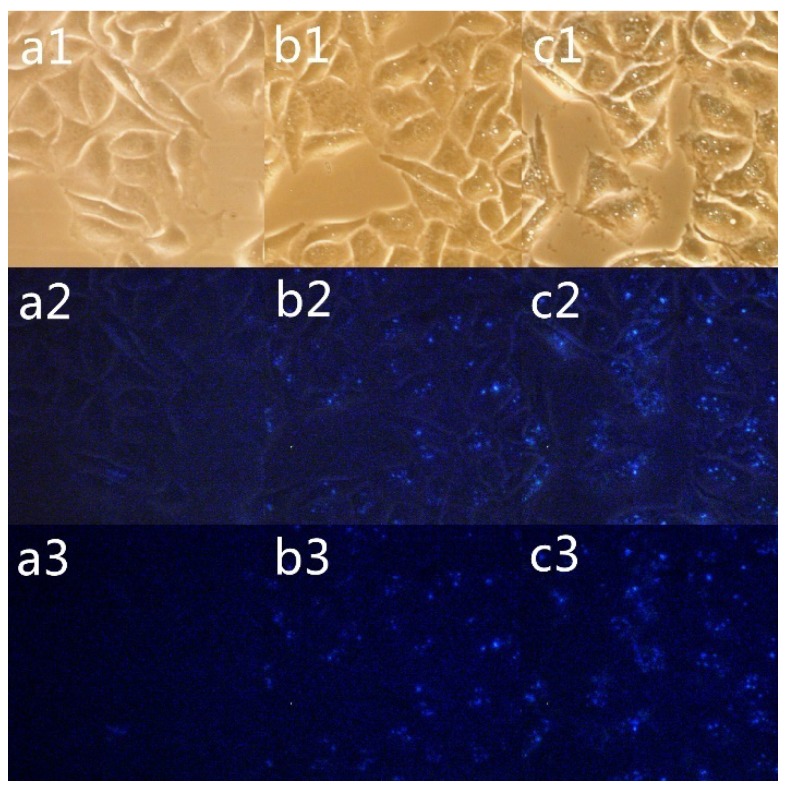
Inverted fluorescent microscope images of SMMC-7721 cells. The cells were incubated with 20 µM CPT (a1–a3), 10 µM E2 (b1–b3), or 20 µM E2 (c1–c3) or for 4 h at 37 °C.

Fluorescence and phase contrast images of SMMC-7721 cells treated with CPT and **E2**were obtained using an inverted fluorescence microscope with ultraviolet light. Compound **E2** was selected for this experiment, because **E2** showed good cytotoxic activity towards SMMC-7721 and low cytotoxicity in MCF-7 cells. Inverted fluorescence microscopy (Figure 1) indicated that **E2** accumulated in the SMMC-7721 cells, as evidenced by the blue-green fluorescence that increased in a concentration-dependent manner. In contrast, cells exposed to CPT showed no distinct fluorescence. The cause of the accumulated blue-green fluorescence in SMMC-7721 is structure of CPT in **E2**, so at same concentration, **E2** showed an better affinity for SMMC-7721 cell than CPT. Moreover, MCF-7 cells exposed to either **E2** or CPT were also imaged (data not shown), but no fluorescent signal in MCF-7 cells was observed with either treatment, so **E2** does not have a good affinity for MCF-7 cells. This result was consistent with our MTT test data. These results suggested that the introduction of DCA by an amide at the CPT 20-hydroxyl position greatly increased the activity of the new compound entering into SMMC-7721 cells.

#### 2.2.3. Lactone Stability

To measure the stability of these novel CPT-bile acid derivatives, Compounds **E2**, **F2**, **G2**, and **H2** were subjected to rat blood serum stability studies (Table 1). Half-life of the tested analogues was all >24 h in rat blood serum, and the lactone stability of the tested analogues was improved significantly compared to CPT (half-life of CPT is 2.3 h). 

#### 2.2.4. *In Vivo* Anti-Tumour Efficacy Studies

Preliminary anti-tumour activity studies of **E2**, **G4**, and **H2** were evaluated *in vivo* in a mouse liver adenocarcinoma model (H_22_) and compared with CPT. An *in vivo* toxicity study showed that the maximum tolerated dose (MTD) values of these CPT-bile acid analogues were >60 mg/kg (data not shown). Three doses of 15 mg/kg, 30 mg/kg, and 45 mg/kg were therefore selected for treatment of the mice by intraperitoneal (i.p.) injection every two days (three doses in all). The data in Table 2 show that the novel CPT-bile acid analogues had significant anti-tumour activity and the low weight loss, compared to CPT group. **G4**, with a tumour inhibitory rate (TIR) of 71.12% at 57.63 µmol/kg (higher than the TIR of 27.45 µmol/kg CPT), showed the best anti-tumour activity. The dose of 27.45 µmol/kg CPT is maximum dose which the mouse can tolerate in this project. Moreover, the effects of **G4**, **E2** and **H2** all showed better dose-dependency over the range tested. Although **E2** had a lower *in vitro* IC_50_ value than CPT in SMMC-7721 cells, it showed poorer anti-tumour activity *in vivo*. Interestingly, in compound **G4**, CPT and CDCA are joined by an ester bond, and **E2** is a conjugate which CPT jointed with DCA by an amide, but **G4** showed superior *in vivo* anti-tumour activity than **E2**. 

**Table 2:** Inhibitory effects of CPT-bile acid analogueson H_22_ mice liver tumours.

Compound	Dose (µmol/kg)	Mice Number		Body Weight (g)		TIR (%)
		Start of Study	End of Study	Start of Study	End of Study	
**Control**	-	4	4	21.3	25.5	-
**CPT**	27.45 × 3	4	3	21.4	18.6	67.26%
**E2**	19.23 × 3	4	4	19.9	21.9	4.09%
	38.45 × 3	4	4	20.5	21.5	16.10%
	57.69 × 3	4	4	20.5	20.6	40.13%
**G4**	19.21 × 3	4	4	19.1	19.4	35.45%
	38.42 × 3	4	4	21.0	21.6	64.55%
	57.63 × 3	4	4	19.8	22.1	71.12%
**H2**	18.82 × 3	4	3	22.1	21.1	13.27%
	37.64 × 3	4	4	21.9	18.2	57.99%
	56.46 × 3	4	3	21.6	20.0	61.19%

TIR, Tumour inhibitory rate.

## 3. Experimental

### 3.1. General Information

All solvents and commercially available reagents were purchased from the suppliers and used without further purification. ^1^H-NMR and ^13^C-NMR spectra were recorded using Bruker DPX 500 (Bruker, Billerica, MA, USA). Spectra were recorded in CDCl_3_ and DMSO solutions and chemical shifts are reported in parts per million (ppm) relative totetramethylsilane (TMS) as the standard. IR spectra were recorded on an Infinity Spectrum One spectrophotometer (Shimadzu, Kyoto, Japan). Mass spectra were recorded on an Agilent 1100 Series VS (ES, 4000 V) mass spectrometer (Applied Biosystems, Toronto, Canada). Melting points were measured using a Büchi B-450 apparatus (Shanghaishenguang, Shanghai, China). Flash chromatography was performed with ACROS silica gel (particle size 0.030–0.040 mm, pore diameter ca. 6 nm) using a glass column.

### 3.2. Compound Synthesis

Synthesis of *Camptothecin-20 (S)-O-(N-(tert-butoxycarbonyl) glycine) Ester* (**1**) and Compound *Camptothecin-20 (S)-O-glycine ester* (**2**)

EDCI (383.4 mg, 2 mmol) and DMAP (36 mg, 0.34 mmol) were added to a solution with CPT (139.2 mg, 0.4 mmol) and *N*-(*tert*-butoxycarbonyl) glycine (140.1 mg, 0.8 mmol) in dichloromethane (15 mL) and stirred at room temperature for 2 h. Chloroform (35 mL) was subsequently added, and the organic phase was washed with water (50 mL) and saturated sodium bicarbonate aqueous solution (50 mL), and then dried using Mg_2_SO_4_ before evaporating to dryness. Compound **1**, a cream-coloured solid was obtained. Total yield 94.8%; M.p. 213.6–214.3 °C; IR (KBr) υ 3315, 3052, 2976, 2936, 2881, 2351, 2320, 1757, 1714, 1652, 1606, 1553, 1500, 1456, 1402, 1392, 1357, 1251, 1157, 1053, 1029, 947, 761, 723 cm^−1^; ^1^H-NMR (500 MHz, CDCl_3_-*d*_6_): δ 4.062–4.108 (1H, d, *J =* 25 Hz, -CH_2_-), 4.195–4.243 (1H, d, *J =* 24 Hz, -CH_2_-), 4.992 (1h, s, -NH-), 5.306 (2H, s, H-17), 5.391–5.426 (1H, d, *J =* 17.5 Hz, H-5), 5.681–5.715 (1H, d, *J =* 17 Hz, H-5), 7.399 (1H, s, H-14), 7.682–7.712 (1H, t, *J =* 15 Hz, H-10), 7.850–7.880 (1H, t, *J =* 15 Hz, H-11), 7.953–7.968 (1H, d, *J =* 7.5 Hz, H-12), 8.302–8.319 (1H, d, *J =* 8.5 Hz, H-9), 8.435 (1H, s, H-7); MS (ESI): *m/z* 506.1 (M+H)^+^. Compound **1** (191.5 mg) was added to a solution of hydrogen chloride/methanol (10 mL). The mixture was stirred at room temperature for 3 h and evaporated to dryness to yield compound **2** as a yellow-coloured solid. Total yield 95%; MS (ESI): *m/z* 441.5 (M+H)^+^. 

Synthesis of *3α,7α,12α-Trihydroxy-5β-cholan-24-oic Acid 2,5-dioxopyrrolidin-1-yl Ester* (**7**)

CA (40.8 mg, 0.1 mmol) was dissolved in DMSO (2 mL). *N*-Hydroxysulphosuccinimide (NHS, 172.5 mg, 1.5 mmol) and EDCI (286.5 mg, 1.5 mmol) were added and the mixture was stirred at room temperature for 2 h. Chloroform (100 mL) was subsequently added and the organic phase was washed with water (100 mL) and saturated sodium bicarbonate aqueous solution (100 mL). The organic solution was dried using Mg_2_SO_4_ and evaporated to dryness. The crude product was separated and purified by column chromatography to obtain a white solid. Yield: 60%; M.p. 103–106 °C; IR (KBr) υ 3377, 3361, 2937, 2968, 1815, 1753, 1658, 1465, 1446, 1435, 1411, 1375, 1296, 1249, 1209, 1112, 1074 1045 cm^−1^; ^1^H-NMR (500 MHz, DMSO-*d*_6_): δ 0.70 (3H, s, H-18), 0.88 (3H, s, H-19), 1.05 (3H, s, H-21), 2.83 (4H, d, *J* = 3.5 Hz, CH_2_), 3.53 (1H, m, 3α-OH), 3.87 (1H, s, 7α-OH), 4.02 (1H, s, 12α-OH); MS (ESI): *m/z*, 528.6 (M+Na)^+^

Synthesis of *3α,12α-Dihydroxy-5β-cholan-24-oic Acid 2,5-dioxopyrrolidin-1-yl Ester* (**8**)

The synthesis was carried out as described for compound **7**, and a white solid was obtained. Yield: 65%; M.p. 181–184 °C; IR (KBr) υ 3363, 2973, 2893, 2868, 1813, 1784, 1743, 1463, 1446, 1436, 1367, 1338, 1205, 1112, 1068, 1045, 1028 cm-1; ^1^H-NMR (500 MHz, DMSO-*d*_6_): δ 0.65 (3H, s, H-18), 0.90 (3H, s, H-19), 1.03 (3H, s, H-21), 2.83–2.85 (4H, d, CH2), 3.63 (1H, m, 3α-OH), 4.07 (1H, s, 12α-OH), MS (ESI): *m/z*, 530.6 (M+H_2_O+Na)^+^. 

Synthesis of *3α,7β-Dihydroxy-5β-cholan-24-oic Acid 2,5-dioxo-pyrrolidin-1-yl Ester* (**9**)

The synthesis was carried out as described for compound **7**, and a white solid was obtained. 

Synthesis of *3α, 7α-Dihydroxy-5β-cholan-24-oic Acid 2,5-dioxopyrrolidin-1-yl Ester* (**10**)

The synthesis was carried out as described for compound **7**, and a white solid was obtained. Yield: 63%; M.p. 241–243 °C; IR (KBr) υ 3412, 2931, 2868, 1815, 1784, 1743, 1732, 1448, 1435, 1413, 1367, 1330, 1289, 1207, 1168, 1128, 1111, 1072, 1047, 1001, 979 cm^−1^; ^1^H-NMR (500 MHz, DMSO-*d*_6_): δ 0.67 (3H, s, H-18), 0.90 (3H, s, H-19), 0.97 (3H, s, H-21), 2.83-2.85 (4H, d, CH_2_), 3.44 (1H, m, 3α-OH), 3.85 (1H, s, 7α-OH); MS (ESI): *m/z* 544.8 (M+Na+CH_3_OH)^+^.

Synthesis of *Camptothecin-20 (S)-O-glycine Ester-[N-(3′α,7′α,12′α-trihydroxy-24′-carbonyl-5′β-cholane)]* (**E1**)

Triethylamine (100 µL) was added to a solution of compound **7** (50.5 mg, 0.1 mmol) and compound **2** (44 mg, 0.1 mmol) in DMSO (2 mL), and the mixture was stirred at room temperature for 2 h (monitored by TLC). Water was then added to the solvent, and a pale yellow precipitate formed immediately. The mixture was filtered, and the solid was washed with water. The crude product was separated and purified by column chromatography to obtain a yellow solid. Yield: 75%; M.p. 200–203 °C; IR (KBr) υ 3419, 3062, 2924, 2858, 2603, 2495, 2308, 1755, 1716, 1660, 1604, 1558, 1504, 1456, 1402, 1377, 1352, 1232, 1076, 1049, 981, 950 cm^−1^; ^1^H-NMR (500 MHz, CDCl_3_-*d*_6_) δ: 0.492 (3H, s, H-18 (CA)), 3.190 (1H, s, H-3 (CA)), 3.550 (1H, s, H-7 (CA)), 3.722 (1H, s, H-12 (CA)), 3.950 (1H, s, 3α-OH(CA)), 3.950–3.985 (1H, d, *J =* 17.5 Hz, -CH2-), 4.038 (1H, s, 7α-OH(CA)), 4.045 (1H, d, -CH2-), 4.280 (1H, s, 12α-OH(CA)), 3.985-5.300 (2H, s, H-17), 5.494 (2H, s, H-5), 7.165 (1H, s, H-14), 7.723–7.738 (1H, t, H-10), 7.875 (1H, t, H-11), 8.127–8.143 (1H, d, *J =* 8 Hz, H-12), 8.173–8.190 (1H, d, *J =* 8.5 Hz, H-9), 8.330 (1H, s, -NH-), 8.702 (1H, s, H-7); MS (ESI): *m/z* 796.7 (M+H)^+^.

Synthesis of *Camptothecin-20 (S)-O-glycine Ester-[N-(3′α,12′α-dihydroxy-24′-carbonyl-5′β-cholane)]* (**E2**)

The synthesis was carried out as described for compound **E1**, and a yellow solid was obtained. Yield: 74%; M.p. 203–204 °C; IR(KBr) υ 3421, 3064, 2927, 2864, 2370, 1751, 1662, 1616, 1560, 1500, 1458, 1402, 1375, 1325, 1232, 1166, 1051, 997, 891 cm^−1^; ^1^H-NMR (500 MHz, CDCl_3_-*d*_6_) δ: 0.567 (3H, s, H-19 (DCA)), 0.842–0.855 (3H, d, *J =* 6.5 Hz, H-21 (DCA)), 0.878 (3H, s, H-18 (DCA)), 3.60 (1H, s, H-3 (DCA)), 4.015 (1H, s, H-12 (DCA)), 4.120–4.166 (1H, t, *J =* 22.4 Hz, -CH2-), 4.437–4.487 (1H, t, *J =* 25 Hz, -CH2-), 5.298 (2H, s, H-17), 5.395–5.429 (1H, m, *J =* 17 Hz, H-5), 5.687–5.721 (1H, m, *J =* 17 Hz, H-5), 5.853 (1H, s, -NH-), 7.262 (1H, s, H-14), 7.681 (1H, t, H-10), 7.848 (1H, t, H-11), 7.936–7.952 (1H, d, *J =* 8 Hz, H-12), 8.252–8.269 (1H, d, *J =* 7 Hz, H-9), 8.401 (1H, s, H-7); MS (ESI): *m/z* 780.5 (M+H)^+^.

Synthesis of *Camptothecin-20 (S)-O-glycine Ester-[N-(3′α,7′β-dihydroxy-24′-carbonyl-5′β-cholane)]* (**E3**)

The synthesis was carried out as described for compound **E1**, and a yellow solid was obtained. Yield: 75%; M.p. 209–211 °C; IR(KBr) υ 3419, 3062, 2927, 2861, 2349, 1755, 1662, 1606, 1558, 1506, 1456, 1402, 1375, 1296, 1232, 1182, 1134, 1051, 1014, 952, 902, 891 cm^−1^; ^1^H-NMR (500 MHz, CDCl_3_-*d*_6_) δ: 0.591 (3H, s, H-18 (UDCA)), 3.551 (1H, s, H-3 (UDCA)), 3.573 (1H, s, H-7 (UDCA)), 4.154-4.162 (1H, m, -CH_2_-), 4.460–4.472 (1H, m, -CH_2_-), 5.311 (2H, s, H-17), 5.390–5.424 (1H, m, *J =* 17 Hz, H-5), 5.683–5.717 (1H, m, *J =* 17.5 Hz, H-5), 5.90 (1H, s, -NH-), 7.403 (1H, s, H-14), 7.710–7.726 (1H, t, H-10), 7.882 (1H, t, H-11), 7.962-7.978 (1H, d, *J =* 8 Hz, H-12), 8.330–8.346 (1H, d, *J =* 8 Hz, H-9), 8.458 (1H, s, H-7); MS(ESI): *m/z* 781.1 (M+H)^+^_._

Synthesis of *Camptothecin-20 (S)-O-glycine Ester-[N-(3′α,7′α-dihydroxy-24′-carbonyl-5′β-cholane)]* (**E4**)

This was carried out as described for compound **E1**, and a yellow solid was obtained.Yield: 76%; M.p. 202–203 °C; IR(KBr) υ 3435, 3062, 2929, 2866, 1755, 1662, 1616, 1558, 1521, 1506, 1456, 1402, 1375, 1296, 1255, 1182, 1058, 976, 959 cm^−1^; ^1^H-NMR (500 MHz, CDCl_3_-*d*_6_) δ: 0.594 (3H, s, H-19 (CDCA)), 3.402 (1H, s, H-3 (CDCA)), 3.802 (1H, s, H-7 (CDCA)), 4.167-4.180 (1H, m, -CH_2_-), 4.432–4.452 (1H, m, -CH_2_-), 5.294–5.302 (2H, d, *J =* 4 Hz, H-17), 5.396–5.430 (1H, m, *J =* 17 Hz, H-5), 5.684–5.718 (1H, m, *J =* 17.5 Hz, H-5), 5.811 (1H, s, -NH-), 7.285 (1H, s, H-14), 7.688 (1H, t, H-10), 7.858 (1H, t, H-11), 7.942–7.958 (1H, d, *J =* 8 Hz, H-12), 8.271–8.288 (1H, d, *J =* 8 Hz, H-9), 8.415 (1H, s, H-7); MS (ESI): *m/z* 780.8 (M+H)^+^.

Synthesis of *Camptothecin-20 (S)-O-(N -(tert-butoxycarbonyl)-(d)-Glu(OtBu)) Ester* (**3**) and *Camptothecin-20 (S)-O-(d-Glu(OMe)) Ester* (**4**)

This was carried out as described for compound **1**, and compound **3**, and a yellow solid was obtained. Yield: 81%; M.p. 178–179 °C; IR (KBr) υ 3419, 3307, 3062, 2924, 2858, 2623, 2495, 2308, 1755, 1660, 1604, 1558, 1504, 1456, 1402, 1377, 1352, 1232, 1076, 1049, 981, 950, 914, 900 cm^−1^; ^1^H-NMR (500 MHz, CDCl_3_-*d*_6_) δ: 4.543 (1H, s, -CH-), 5.099 (1H, s,-NH-), 5.276 (2H, s, H-17), 5.368–5.402 (1H, d, *J =* 17 Hz, H-5), 5.687–5.721 (1H, d, *J =* 17 Hz, H-5), 7.408 (1H, s, H-14), 7.647–7.676 (1H, t, *J =* 14.5 Hz, H-10), 7.809–7.839 (1H, t, *J =* 15 Hz, H-11), 7.920–7.936 (1H, d, *J =* 8 Hz, H-12), 8.261–8.279 (1H, d, *J =* 9 Hz, H-9), 8.376 (1H, s, H-7); MS (ESI): *m/z* 634.4 (M+H)^+^. Compound **3(**191.5 mg) was added to hydrogen chloride/methanol (10 mL) and the mixture was stirred at room temperature for 12 h (monitored by TLC). Chloroform (50 mL) was subsequently added, and the organic phase was washed with water (50 mL). The organic solution was dried (Mg_2_SO_4_) and evaporated to dryness, producing compound **4**, a yellow solid. Total yield 90%; MS (ESI): *m/z* 527.5 (M+H)^+^.

Synthesis of *Camptothecin-20 (S)-O-(d-Glu (OMe)) Ester-[N-(3′α,7′α,12′α-trihydroxy-24′-carbonyl-5′β-cholane)]* (**F1**)

This was carried out as described for compound **E1**, and a yellow solid was obtained. Yield: 70%; M.p. 195–196 °C; IR (KBr) υ 3431, 3057, 2926, 2856, 1747, 1662, 1604, 1456, 1402, 1375, 1352, 1232, 1166, 1195, 1049, 1016, 979 cm^−1^; ^1^H-NMR (500 MHz, CDCl_3_-*d*_6_) δ: 0.547 (3H, s, H-19 (CA)), 3.094–3.119 (2H, q, *J =* 12.5 Hz, H-3 (Glu)), 2.556–2.571 (2H, d, *J =* 7.5 Hz, H-4 (Glu)), 3.46 (1H, s, H-3 (CA)), 3.762 (3H, s, H-1 (CH_3_)), 3.762 (1H, s, H-7 (CA)), 3.849 (1H, s, H-3 (CA)), 4.96 (1H, s, H-2 (Glu)), 5.286 (2H, s,H-17), 5.374–5.408 (1H, d, *J =* 17 Hz,H-5), 5.681–5.715 (1H, d, *J =* 17 Hz, H-5), 6.56 (1H, s, H-1 (NH)), 7.419 (1H, s, H-14), 7.690 (1H, t, H-10), 7.856 (1H, t, H-11), 7.926–7.942 (1H, d, *J =* 8 Hz, H-12), 8.310–8.327 (1H, d, *J =* 8.5 Hz, H-9), 8.415 (1H, s, H-7); MS (ESI): *m/z* 882.5 (M+H)^+^.

Synthesis of *Camptothecin-20 (S)-O-(d-Glu(OMe))Ester-[N-(3′α,12′α-dihydroxy-24′-carbonyl-5′β-cholane)]* (**F2**)

This was carried out as described for compound **E1**, and a yellow solid was obtained. Yield: 75%; M.p. 199–200 °C; IR (KBr) υ 3438, 3059, 2930, 2860, 1747, 1662, 1610, 1556, 1402, 1365, 1304, 1258, 1169, 1120,1086, 1049, 1014, 983 cm^−1^; ^1^H-NMR (500 MHz, CDCl_3_-*d*_6_) δ: 0.486 (3H, s, H-19 (DCA)), 0.780 (3H, s, H-21), 2.568–2.594 (2H, t, *J =* 13 Hz, H-4 (Glu)), 3.502 (1H, s, H-3 (DCA)), 3.576 (1H, s, H-12 (DCA)), 3.707 (3H, s, H-6 (CH_3_)), 4.882–4.894 (1H, d, *J =* 6 Hz, H-2 (Glu)), 5.283 (2H, s, H-17), 5.372–5.406 (1H, d, *J =* 17, H-5), 5.680–5.714 (1H, d, *J =* 17 Hz, H-5), 6.279–6.292 (1H, d, *J =* 6.5 Hz, H-6 (N-H)), 7.363 (1H, s, H-14), 7.671 (1H, t, H-10), 7.841 (1H, t, H-11), 7.926–7.942 (1H, d, *J =* 8 Hz, H-12), 8.289–8.306 (1H, d, *J =* 8.5 Hz, H-9), 8.393 (1H, s, H-7); MS (ESI): *m/z* 866.9 (M+H)^+^.

Synthesis of *Camptothecin-20 (S)-O-(d-Glu(OMe)) Ester-[N-(3′α,7′β-dihydroxy-24′-carbonyl-5′β-cholane)]* (**F3**)

This was carried out as described for compound **E1**, and a yellow solid was obtained. Yield: 72%; M.p. 187–189 °C; IR (KBr) υ 3421, 3059, 2929, 2864, 2364, 1747, 1662, 1616, 1558, 1521, 1502,1452,1381,1360,1232,1166,1083,1051,1014,952 cm^−1^; ^1^H-NMR (500 MHz, CDCl_3_-*d*_6_) δ: 0.492 (3H, s, H-19 (UDCA)), 0.660 (3H, s, H-21 (UDCA)), 2.564–2.594 (2H, t, *J =* 15 Hz, H-4 (Glu)), 3.56 (1H, s, H-3 (CA)), 3.622 (1H, s, H-17 (UDCA)), 3.707 (3H, s, H-6 (CH_3_)), 4.860 (1H, s, H-2 (Glu)), 5.290 (2H, s, H-17), 5.376–5.411 (1H, d, *J =* 17.5 Hz, H-5), 5.684–5.718 (1H, d, *J =* 17 Hz, H-5), 6.25 (1H, d, H-6 (NH)), 7.369 (1H, s, H-14), 7.679 (1H, t, H-10), 7.864 (1H, t, H-11), 7.933–7.949 (1H, d, *J =* 8 Hz, H-12), 8.296–8.313 (1H, d, *J =* 7.5 Hz, H-9), 8.402 (1H, s, H-7); MS (ESI): *m/z* 866.9 (M+H)^+^.

Synthesis of *Camptothecin-20 (S)-O-(d-Glu(OMe)) Ester-[N-(3′α 7′α-dihydroxy-24′-carbonyl-5′β-cholane)]* (**F4**)

This was carried out as described for compound **E1**, and a yellow solid was obtained. Yield: 74%; M.p. 182–185 °C; IR(KBr) υ 3566, 3057, 2921, 2851, 2306, 1717, 1662, 1604, 1558, 1506, 1156, 1375, 1325, 1296, 1259, 1232, 1166, 1078, 1051, 1001,989,800 cm^−1^; ^1^H-NMR (500 MHz, CDCl_3_-*d*_6_) δ: 0.513 (3H, s, H-19 (CDCA)), 0.788 (3H, s, H-21 (CDCA)), 2.563–2.592 (2H, t, *J =* 14.5 Hz, H-4 (Glu)), 3.454 (1H, s, H-3 (CDCA)), 3.705 (3H, s, H-6 (CH_3_)), 3.764 (1H, s, H-7 (CA)), 4.888–4.899 (1H, d, *J =* 5.5, H-2 (Glu)), 5.283 (2H, s, H-17), 5.375–5.410 (1H, d, *J =* 17.5 Hz, H-5), 5.683–5.717 (1H, d, *J =* 17 Hz, H-5), 6.26 (1H, d, H-6 (NH)), 7.362 (1H, s, H-14), 7.673–7.688 (1H, t, *J =* 7.5 Hz, H-10), 7.831–7.845 (1H, t, *J =* 7 Hz, H-11), 7.926–7.942 (1H, d, *J =* 8 Hz, H-12), 8.290–8.307 (1H, d, *J =* 8.5 Hz, H-9) 8.393 (1H, s, H-7); MS (ESI): *m/z* 866.9 (M+H)^+^.

Synthesis of *Camptothecin-20 (S)-O-bromoacetate* (**5**)

This was carried out as described for compound **1**, and a yellow solid was obtained. Yield: 97%; M.p. 234–236 °C; IR (KBr) υ 3449, 2970, 1754, 1661, 1558, 1454, 1232, 1151, 1052, 990, 787, 761 cm^−1^; ^1^H-NMR (500 MHz, CDCl_3_-*d*_6_) δ: 1.00 (3H, t, *J =* 7.2 Hz, H-18), 2.16-2.37 (2H, m, H-19), 4.28 (2H, q, CH_2_), 5.31 (2H, s, H-5), 5.43 (1H, d, *J =* 17.5 Hz, H-17), 5.71 (1H, d, *J=* 17.2 Hz, H-17), 7.34 (1H, s, H-14), 7.70 (1H, t, *J =* 7.2 Hz, H-10), 7.87 (1H, t, *J =* 7.2 Hz, H-11), 7.97 (1H, d, *J =* 8.4 Hz, H-12), 8.28 (1H, d, *J =* 8.5 Hz, H-9), 8.45 (1H, s, H-7); MS (ESI): *m/z* 469.4 (M+H)^+^.

Synthesis of *Camptothecin-20 (S)-O-acetate-[N-(3′α,7′α,12′α-trihydroxy-24′-carbonyl-5′β-cholane)]* (**G1**)

Triethylamine (50 µL) was added to a solution of compound **5** (46.9 mg, 0.1 mmol) and CA (81.6 mg, 0.2 mmol) in *N*,*N*-dimethylformamide (DMF) (1 mL), and the mixture was stirred at room temperature for 12 h (monitored by TLC). Chloroform (50 mL) was subsequently added and the organic phase was washed with water (50 mL) before drying (Mg_2_SO_4_) and evaporation to dryness. The crude product was separated and purified by column chromatography, producing a yellow solid. Yield: 62%; M.p. 230–233 °C; IR(KBr) υ 3460, 3062, 2926, 2861, 2345, 2322, 1751, 1666, 1618, 1560, 1506, 1458, 1402, 1379, 1296, 1254, 1232, 1192, 1149, 1076, 1049, 1018, 999, 981, 912 cm^−1^; ^1^H-NMR (500 MHz, CDCl_3_-*d*_6_) δ: 0.624 (3H, s, H-19 (CA)), 3.421 (1H, s, H-3 (CA)), 3.801 (1H, s, H-7 (CA)), 3.917 (1H, s, H-12 (CA)), 4.767–4.881 (2H, q, *J =* 57 Hz, H-1 (CH_2_)), 5.301 (2H, s, H-17), 5.398–5.443 (1H, d, *J =* 17.5 Hz, H-5), 5.674–5.708 (1H, d, *J =* 17 Hz, H-5), 7.355 (1H, s, H-14), 7.689 (1H, t, H-10), 7.854 (1H, t, H-11), 7.946–7.963 (1H, d, *J =* 8.5 Hz, H-12), 8.279–8.296 (1H, d, *J =* 8.5 Hz, H-9) 8.425 (1H, s, H-7); MS (ESI): *m/z* 797.7 (M+H)^+^.

Synthesis of *Camptothecin-20 (S)-O-acetate-[N-(3′α,12′α-dihydroxy-24′-carbonyl-5′β-cholane)]* (**G2**)

This was carried out as described for compound **G1**, and a yellow solid was obtained. Yield: 63%; M.p. 221–224 °C; IR(KBr) υ 3550, 3421, 3089, 2935, 2862, 2349, 1749, 1666, 1616, 1560, 1500, 1456, 1402, 1379, 1365, 1352, 1298, 1230, 1085, 1043, 945, 923 cm^−1^; ^1^H-NMR (500 MHz, CDCl_3_-*d*_6_) δ: 0.610 (3H, s, H-19 (DCA)), 3.600 (1H, s, H-3 (DCA)), 3.913 (1H, s, H-12 (DCA)), 4.769–4.882 (2H, q, *J =* 56.5 Hz, H-1 (CH_2_)), 5.304 (2H, s, H-17), 5.397–5.432 (1H, d, *J =* 17.5 Hz, H-5), 5.670–5.705 (1H, d, *J =* 17 Hz, H-5), 7.365 (1H, s, H-14), 7.699–7.715 (1H, t, *J =* 8 Hz, H-10), 7.862 (1H, t, H-11), 7.956–7.972 (1H, d, *J =* 8 Hz, H-12), 8.294 (1H, d, *J =* 8.5 Hz, H-9), 8.440 (1H, s, H-7); MS (ESI): *m/z* 781.8 (M+H)^+^.

Synthesis of *Camptothecin-20 (S)-O-acetate-[N-(3′α,7′β-dihydroxy-24′-carbonyl-5′β-cholane)]* (**G3**)

This was carried out as described for compound **G1**, and a yellow solid was obtained. Yield: 62%; M.p. 229–230 °C; IR(KBr) υ 3504, 3446, 3064, 2929, 2861, 2315, 1755, 1670, 1618, 1560, 1500, 1456, 1404, 1298, 1232, 1151, 1101, 1051, 1014, 948, 927 cm^−1^; ^1^H-NMR (500 MHz, CDCl_3_-*d*_6_) δ: 0.592 (3H, s, H-19 (UDCA)), 3.568 (2H, s, H-3, H-7 (UDCA)), 4.738–4.771 (1H, d, *J =* 17.5, H-1 (CH2)), 4.851–4.884 (1H, d, *J =* 16.5 Hz, H-1 (CH_2_)), 5.293 (2H, s, H-17), 5.395–5.430 (1H, d,*J =* 17.5 Hz, H-5), 5.670–5.705 (1H, d, *J =* 17.5 Hz, H-5) 7.281 (1H, s, H-14), 7.678–7.693 (1H, t, *J =* 7.5 Hz, H-10), 7.842–7.845 (1H, t, *J =* 1.5 Hz, H-11), 7.940–7.956 (1H, d, *J =* 8 Hz,H-12), 8.242–8.259 (1H, d, *J =* 8.5 Hz,H-9), 8.400 (1H, s, H-7); MS (ESI): *m/z* 781.8 (M+H)^+^.

Synthesis of *Camptothecin-20 (S)-O-acetate-[N-(3′α,7′α-dihydroxy-24′-carbonyl-5′β-cholane)]* (**G4**)

This was carried out as described for compound **G1**, and a yellow solid was obtained. Yield: 63%; M.p. 228–229 °C; IR (KBr) υ 3564, 3481, 3064, 2926, 2854, 2347, 1755, 1670, 1620, 1560, 1500, 1458, 1404, 1381, 1354, 1232, 1203, 1147, 1011, 979 cm^−1^; ^1^H-NMR (500 MHz, CDCl_3_-*d*_6_) δ: 0.592 (3H, s, H-19 (CDCA)), 3.454 (1H, s, H-3 (CDCA)), 3.800 (1H, s, H-7 (CDCA)), 4.746-4.779 (1H, d, *J =* 16.5 Hz, H-1 (CH_2_)), 4.845–4.878 (1H, d, *J =* 16.5 Hz, H-1 (CH_2_)), 5.288 (2H, s, H-17), 5.397–5.431 (1H, d, *J =* 17 Hz, H-5), 5.668–5.702 (1H, d, *J =* 17 Hz, H-5), 7.290 (1H, s, H-14), 7.680 (1H, t, H-10), 7.843 (1H, t, H-11), 7.940–7.956 (1H, d, *J =* 2 Hz, H-12), 8.248–8.265 (1H, d, *J =* 8.5 Hz, H-9), 8.404 (1H, s, H-7); MS (ESI): *m/z* 781.9 (M+H)^+^.

Synthesis of *10-O-(3-Bromopropyl)-20(S)-camptothecin* (**6**)

To a solution of 10-hydroxy CPT (182 mg, 0.5 mmol) and potassium carbonate (345.94 mg, 2.5 mmol) in DMF (1.5 mL) 1,3-dibromopropane (100 µL) was added, and the mixture was stirred at 60 °C for 10 h under nitrogen conditions (monitored by TLC). Water was then added to the solvent, and a pale yellow precipitate appeared immediately. The mixture was filtered and the solid was washed with water. The crude product was separated and purified by column chromatography to obtain a yellow solid. Yield: 85%; M.p. 235–237 °C; IR (KBr) υ 3398, 2955, 2925, 1747,1660, 1595, 1556, 1508, 1429, 1370, 1237, 1147, 1105, 1049, 1015, 532 cm^−1^; ^1^H-NMR (500 MHz, DMSO-*d*_6_) δ: 0.88 (3H, t, *J =* 7.4 Hz, H-18), 1.86 (2H, m, H-19), 2.36 (2H, t, *J =*5.6 Hz, CH_2_), 3.74 (2H, t, *J =* 5.6 Hz, CH_2_), 4.26 (2H, t, *J =* 5.6 Hz, CH_2_), 5.25 (2H, s, H-5), 5.41 (2H, s, H-17), 6.53 (1H, s, 20-OH), 7.27 (1H, s, H-14), 7.50 (1H, d, *J =* 10 Hz, H-11), 7.55 (1H, s, H-9), 8.05 (1H, d, *J =* 10 Hz, h-12), 8.53 (1H, s, H-7); MS (ESI): *m/z* 485.0 (M+H)^+^.

Synthesis of *10-O-(3-O-(3′α,7′α,12′α-Trihydroxy-24′-carbonyl-5′β-cholan)-propyl)-20(S)-camptothecin* (**H1**)

CA (81.6 mg, 0.2 mmol) was added to a solution of compound **6** (47 mg, 0.1 mmol) and potassium carbonate (34.5 mg, 0.25 mmol) in 1 mL DMF, and the mixture was stirred at 60 °C for 10 h under nitrogen conditions (monitored by TLC). Water was then added to the solvent, and a pale yellow precipitate appeared immediately. The mixture was filtered and the solid was washed with water. The crude product was separated and purified by column chromatography to obtain a yellow solid. Yield: 73%; M.p. 195–200 °C; IR (KBr) υ 3419, 3095, 2926, 2866, 2349, 1739, 1658, 1622, 1597, 1558, 1504, 1463, 1371, 1296, 1238, 1157, 1047, 912cm^−1^; ^1^H-NMR (500 MHz, CDCl_3_-*d*_6_) δ: 0.598 (3H, s, H-19), 3.399 (1H, s, H-3 (CA)), 3.720 (1H, s, H-7 (CA)), 3.86 (1H, s, H-20), 3.926 (1H, s, H-12 (CA)), 4.26 (2H, t, H-1 (CH_2_)), 4.329–4.353 (2H, t, *J =* 12 Hz, H-2 (CH_2_)), 5.324–5.348 (1H, d, *J =* 12 Hz, H-5), 5.348 (2H, s, H-17), 5.712–5.745 (1H, d, *J =* 16.5 Hz, H-5), 7.176 (1H, s, H-14), 7.460–7.484 (1H, d, *J =* 12 Hz, H-11), 7.656 (1H, s, H-9), 8.132–8.150 (1H, d, *J =* 9 Hz, H-12), 8.274 (1H, s, H-7); MS (ESI): *m/z* 813.9 (M+H)^+^.

Synthesis of *10-O-(3-O-(3′α,12′α-Dihydroxy-24′-carbonyl-5′β-cholan)-propyl)-20(S)-camptothecin* (**H2**)

This was carried out as described for compound **H1**, and a yellow solid was obtained. Yield: 75%; M.p. 193–197 °C; IR (KBr) υ 3400, 3097, 2926, 2858, 2349, 1739, 1658, 1622, 1595, 1556, 1504, 1446, 1371, 1296, 1238, 1155, 1095, 1045, 968, 912 cm^−1^; ^1^H-NMR (500 MHz, DMSO-*d*_6_) δ: 0.316 (3H, s, H-19), 3.611 (1H, s, H-3 (DCA)), 4.120–4.127 (1H, s, H-7 (DCA)), 4.227 (4H, s, H-1, H-2 (CH_2_)), 4.227 (1H, s, OH-3 (DCA)), 4.417–4.426 (1H, s, OH-7 (DCA)), 5.275 (2H, s, H-17), 5.417 (2H, s, H-5), 6.498 (1H, s, OH-20), 7.295 (1H, d, H-14), 7.532 (2H, d, H-11, H-9), 8.061–8.079 (1H, d, *J =* 9 Hz, H-12), 8.542 (1H, s, H-7); MS (ESI): *m/z* 797.7 (M+H)^+^.

Synthesis of *10-O-(3-O-(3′α,7′β-Dihydroxy-24′-carbonyl-5′β-cholan)-propyl)-20(S)-camptothecin* (**H3**)

This was carried out as described for compound **H1**, and a yellow solid was obtained. Yield: 73%; M.p. 201–205 °C; IR (KBr) υ 3350, 3095, 2926, 2858, 2357, 1739, 1660, 1600, 1556, 1504, 1454, 1371, 1238, 1157, 1103, 1047, 1012, 964, 900, 827 cm-1; 1H-NMR (500 MHz, CDCl3-*d*_6_) δ: 0.595 (3H, s, H-19 (UDCA)), 3.456–3.461 (1H, s, H-3 (UDCA)), 3.578 (1H, s, H-12 (UDCA)), 3.857 (1H, s, OH-20), 4.227 (2H, t, H-1 (CH2)), 4.338 (2H, t, H-2 (CH2)), 5.293 (2H, s, H-17), 5.293–5.325 (1H, d, *J =* 16 Hz, H-5), 5.729–5.761 (1H, d, *J =* 16 Hz, H-5), 7.164 (1H, s, H-14), 7.462–7.483 (1H, d, *J =* 11 Hz, H-11), 7.648 (1H, s, H-9), 8.129–8.147 (1H, d, *J =* 8 Hz, H-12), 8.251 (1H, s, H-7); MS (ESI): *m/z* 797.9 (M+H)^+^.

Synthesis of *10-O-(3-O-(3′α,7′α-Dihydroxy-24′-carbonyl-5′β-cholan)-propyl)-20(S)-camptothecin* (**H4**)

This was carried out as described for compound **H1**, and a yellow solid was obtained. Yield: 71%; M.p. 202–205 °C; IR (KBr) υ 3093, 2924, 2854, 2351, 1732, 1660, 1624, 1558, 1506, 1456, 1373, 1240, 1157, 1103, 1047, 1001, 977, 945, 825cm^−1^; ^1^H-NMR (500 MHz, CDCl_3_-*d*_6_) δ: 0.583 (3H, s, H-19 (CDCA)), 3.434 (1H, s, H-3 (CDCA)), 3.759 (1H, s, H-12 (CDCA)), 3.764 (1H, s, OH-20), 4.231-4.238 (2H, d, *J =* 3.5 Hz, H-1 (CH_2_)), 4.337–4.340 (2H, d, *J =* 1.5 Hz, H-2 (CH_2_)), 5.301 (2H, s, H-17), 5.301–5.339 (1H, d, *J =* 19 Hz, H-5), 5.724–5.757 (1H, d, *J =* 16.5 Hz, H-5), 7.168 (1H, s, H-14), 7.470–7.489 (1H, d, *J =* 9.5 Hz, H-11), 7.667 (1H, s, H-9), 8.144–8.172 (1H, d, *J =* 13 Hz, H-12), 8.256 (1H, s, H-7); MS (ESI): *m/z* 797.6 (M+H)^+^.

### 3.3. MTT Assay

The cytotoxicity of these compounds against three cancer cell lines was assayed using a conventional microtitre plate tetrazolium cytotoxicity assay. The human hepatoma cancer cell line (SMMC-7721), the colon cancer cell line (HCT-116), and the human breast cancer cell line (MCF-7) were provided by The Chinese Academy of Sciences cell bank. These cells were plated at a density of 1 × 10^5^ cells/mL and incubated in a 96-well late for 24 h (37 °C, 5% CO_2_). The cells were then exposed to various concentrations of the compounds (0.0032, 0.016, 0.08, 0.4, 2, 10, and 50 µM/mL) continuously for 72 h. After this incubation, MTT solution (20 μL, 5 mg/mL) was added to each well and incubated for a further 4 h before removing the media. Formazan crystals were then dissolved by the addition of DMSO (100 μL per well). After mixing, the absorbance was read at 540 nm on a microplate reader. These assays were performed in three independent experiments. Wells containing no drugs, or CPT, were used as negative and positive controls, respectively. The IC_50_ values, defined as the concentration of compounds that produced a 50% decrease in surviving cells, were calculated using the Logit-method.

### 3.4. Fluorescence and Phase Contrast Microscopy

Compound **E2** (final concentrations of 10 µM/mL and 20 µmol/mL) and CPT (final concentrations of 10 µM/mL and 20 µM/mL) were directly added to the cell culture medium and incubated for 1 h, 2 h or 4 h at 37 °C. The cells were then gently washed three times with physiological saline. Cellular fluorescence was imaged using a fluorescence microscope (Zeiss inverted Microscope Axiovert 200 equipped with a NIKON DS-FiL (Sendai, Japan). The images were taken using ultraviolet light. 

### 3.5. Lactone Stability in Serum

Compounds **E2**, **F2**, **G2**, and **H2** were dissolved in DMSO (100 μL, 4 mM/L) and incubated in rat blood serum (4 mL) at 37 °C. Aliquots were taken at different time points (0, 2, 4, 6, 8, 10, 12, and 24 h) and examined by HPLC, using CPT as the reference.

### 3.6. In Vivo Anti-Tumour Activity

Anti-tumour activity screens of CPT-bile acid analogues were conducted in KM mice with H_22_ mouse liver tumours. Briefly, female mice weighing 18–22 g were subcutaneously inoculated at the right flank with a tumour cell suspension (1 × 10^6^ cells) in 0.2 mL of phosphate-buffered saline. The mice were divided into subgroups 24 h after inoculation. These groups (*n* = 4) were treated with either vehicle (control), CPT, or the CPT-bile acid analogues. Mice received i.p. treatments once every two days. When the tumour weight of the control group reached 1.0–1.5 g, the mice were killed and the TIR (%) calculated.

## 4. Conclusions

Sixteen different CPT-bile acid analogues were synthesised using different bile acids and linker chains. To explore the effects of this on CPT activity, the anti-tumour activities of these analogues were evaluated *in vitro* and *in vivo*. Most of the CPT-bile acid analogues showed greater cytotoxicity in a human hepatoma cell line (SMMC-7721) than in a breast cancer cell line (MCF7), with the exception of **H1**–**H4**. Compounds **E2** and **G4** showed better inhibition of the cancer cells than CPT. Fluorescent accumulation of **E2** was detected in the SMMC-7721 cells and **E2** entered this cell line more effectively than it entered MCF-7 cells. The lactone stability of the analogues tested in rat blood serum generally increased, indicating a more stable lactone character than CPT. *In vivo*, the use of an ester bond to connect a bile acid with CPT (**G4**) produced the best anti-tumour activity. Taken together, these findings indicated that introducing a bile acid group at the 20-hydroxyl group of CPT affected drug activity more than introduction at the 10-hydroxyl group. The results of this study suggested that CPT-bile acid analogues are promising candidates for anti-cancer therapy. 
